# Differentiation of pseudoprogression and real progression in glioblastoma using ADC parametric response maps

**DOI:** 10.1371/journal.pone.0174620

**Published:** 2017-04-06

**Authors:** Caroline Reimer, Katerina Deike, Markus Graf, Peter Reimer, Benedikt Wiestler, Ralf Omar Floca, Philipp Kickingereder, Heinz-Peter Schlemmer, Wolfgang Wick, Martin Bendszus, Alexander Radbruch

**Affiliations:** 1 Department of Neuroradiology, University of Heidelberg Medical Center, Heidelberg, Germany; 2 Department of Radiology, Deutsches Krebsforschungszentrum (DKFZ), Heidelberg, Germany; 3 Institute of Diagnostic and Interventional Radiology, Klinikum Karlsruhe, Academic Teaching Hospital of the University of Freiburg, Karlsruhe, Germany; 4 Department of Neuroradiology, Technical University Munich, Munich, Germany; 5 Department of Neurology, University of Heidelberg Medical Center, Heidelberg, Germany; 6 Department of Diagnostic and Interventional Radiology and Neuroradiology, University Hospital Essen, University of Duisburg-Essen, Essen, Germany; 7 German Cancer Consortium (DKTK), Deutsches Krebsforschungszentrum (DKFZ), Heidelberg, Germany; Julius-Maximilians-Universität Würzburg, GERMANY

## Abstract

**Purpose:**

The purpose of this study was to investigate whether a voxel-wise analysis of apparent diffusion coefficient (ADC) values may differentiate between progressive disease (PD) and pseudoprogression (PsP) in patients with high-grade glioma using the parametric response map, a newly introduced postprocessing tool.

**Methods:**

Twenty-eight patients with proven PD and seven patients with PsP were identified in this retrospective feasibility study. For all patients ADC baseline and follow-up maps on four subsequent MRIs were available. ADC maps were coregistered on contrast enhanced T1-weighted follow-up images. Subsequently, enhancement in the follow-up contrast enhanced T1-weighted image was manually delineated and a reference region of interest (ROI) was drawn in the contralateral white matter. Both ROIs were transferred to the ADC images. Relative ADC (rADC) (baseline)/reference ROI values and rADC (follow up)/reference ROI values were calculated for each voxel within the ROI. The corresponding voxels of rADC (follow up) and rADC (baseline) were subtracted and the percentage of all voxels within the ROI that exceeded the threshold of 0.25 was quantified.

**Results:**

rADC voxels showed a decrease of 59.2% (1^st^ quartile (Q1) 36.7; 3^rd^ quartile (Q3) 78.6) above 0.25 in patients with PD and 18.6% (Q1 3.04; Q3 26.5) in patients with PsP (*p* = 0.005). Receiver operating characteristic curve analysis showed the optimal decreasing rADC cut-off value for identifying PD of > 27.05% (area under the curve 0.844±0.065, sensitivity 0.86, specificity 0.86, *p* = 0.014).

**Conclusion:**

This feasibility study shows that the assessment of rADC using parametric response maps might be a promising approach to contribute to the differentiation between PD and PsP. Further research in larger patient cohorts is necessary to finally determine its clinical utility.

## Introduction

Median overall survival (OS) of patients with glioblastoma is still limited to 12–18 months [1–3]. Standard therapy includes a concordant chemoradiation therapy (CRT) followed by six cycles of adjuvant chemotherapy using temozolomide as chemotherapeutic substance [1].

Treatment response assessment is often challenging due to the appearance of an imaging phenomenon coined pseudoprogression (PsP). PsP refers to a new or increasing area of gadolinium contrast enhancement on T1-weighted (T1w) magnetic resonance imaging (MRI) studies that appears mainly within 3 months after completion of radiochemotherapy and which subsequently subsides without any change in therapy [4,5]. Different studies have reported an incidence of PsP between 10–40% [5–11].

Even though studies reported an increased diagnostic accuracy for the differentiation of PsP and progressive disease (PD) using advanced MRI techniques, no technique has been proven to reliably differentiate between PsP and PD [12–19]. According to the Response Assessment in Neuro-Oncology (RANO) criteria, published in 2010, patients with new or increased contrast enhancement within the first 12 weeks after radiotherapy may be excluded from further treatment or clinical trials for recurrent therapy unless the enhancement is outside the radiation field or histopathological confirmation is available. Otherwise, the diagnosis is established on the next follow-up scan [4]. As highlighted by Radbruch et al, this approach can result in the delayed treatment of patients with the most aggressive tumors that tend to recur early [5]. Therefore advanced imaging techniques that can provide a reliable differentiation between PsP and PD are obviously needed.

Generally, diffusion weighted MRI (DWI) has been proposed as an early imaging biomarker for tumor response [6]. Increased diffusion of water molecules is measured as an increase in the apparent diffusion coefficient (ADC) occuring shortly after successful treatment. The increase in ADC presumably reflects disintegration of cellular membranes, reduction in cell density and as a result an increase in extracellular space [20].

A major limitation of recent studies dealing with diffusion-based MRI techniques was the use of a Region of Interest (ROI) approach. This ROI approach does not reflect the enormous heterogeneity of glioblastoma. Variances between different regions of the glioblastoma might be neglected if this approach is used, due to the mean values that are calculated within the ROI. To overcome this limitation, the parametric response map, a novel postprocessing tool, that is based on a voxel-wise analysis, was introduced [20–24].

The objective of the current study was to determine whether voxel-wise ADC changes calculated in parametric response maps can differentiate between PsP and PD in glioblastoma patients.

## Methods

### Patients

This retrospective study was approved by the institutional review board (ethical commission University of Heidelberg S-320/2012). Due to the retrospective nature and the poor prognosis of glioblastoma patients, written consent was waived by the institutional review board. The whole study was carried out using anonymized data. Data are available from the corresponding author for researchers who meet the criteria for access to confidential data. Subsequently treated patients were identified based on the histopathologically proven diagnosis of a glioblastoma during the period of January 1, 2007 and August 31, 2012. The patient cohort in this study is based on a previous study conducted by Radbruch et al. with 79 patients being enrolled.[25] The hospital picture archiving and communication system (Centricity PACS, version 3.0.4, GE, Healthcare Integrated IT Solutions, Barrington, IL) was reviewed for these patients' postoperative (= baseline) MRI scans and follow-up scans. In the precursive study only registered patients treated with standard CRT according to Stupp et al. [1], with a minimum age of 18 years, a postoperative baseline scan within 72 h after surgery as well as regular MRI scans conducted until an enhancement increase on T1w MRI had been included. Moreover patients had to present a contrast enhancement increase of at least 25% of an original lesion with ≥ 10 mm of perpendicular diameters or a new nodular component ≥ 10 mm within the radiation field in the first, second, third or fourth follow-up compared with the baseline scan. For this study DWI was requested in addition to conventional contrast-enhanced T1w MRI. Patients with a substantial mass effect with change of tumor position had to be excluded from the study. Finally, 35 patients with newly diagnosed histological proven glioblastoma met the outlined criteria (Table 1).

**Table 1:** Patient characteristic.

Characteristic	Total	PD	PsP
Total n of patients	35	28	7
Age, years			
Median	60	54	62.5
Q1, Q3	50, 60	50.5, 67.5	50, 61.5
Range	20–79	20–79	38–68
Mean	58.2	59.1	54.7
SD	±12.4	±12.9	±10.1
Gender, n (%)			
Male	26 (74.3)	23 (82.1)	3 (42.9)
Female	9 (25.7)	5 (17.9)	4 (57.1)
Pathology, n			
WHO Grade 4	35	28	7
IDH mutation status, n			
Positive	0	0	0
Negative	26	22	4
Unknown	9	6	3
Location, n (%)			
Frontal/ Temporal	33 (94.3)	26 (92.9)	7 (100)
Parietal	2 (5.7)	2 (7.1)	0 (0)
KPS (%)			
Median	90	90	90
Q1, Q3	90, 90	90, 100	85, 90
Range	70–100	70–100	80–100
Surgery, n (%)			
Biopsy	2 (5.7)	1 (3.6)	1 (14.3)
Subtotal	7 (20.0)	7 (25.0)	1 (14.3)
Near GTR	26 (74.3)	20 (71.4)	5 (71.4)
Postoperative therapy, n			
RT, any regimen	35	28	7
CT containing TMZ	30	23	7
CT without TMZ	2	2	0
No CT	3	3	0

*PD* Progressive disease, *PsP* Pseudoprogression, *n* number, *Q1* 1^st^ quartile, *Q3* 3^rd^ quartile, *SD* standard deviation, *WHO* World Health Organization, *IDH* Isocitrate dehydrogenase, *KPS* Karnofsky Performance Score, *GTR* Gross total resection, *RT* radiation therapy, *CT* chemotherapy, *TMZ* Temozolomide.

### MRI parameters

Images were acquired using a 3 Tesla MR system (Magnetom Verio/Trio TIM, Siemens Healthcare, Erlangen, Germany) or a 1.5 Tesla MR system (Symphony, Siemens Healthcare, Erlangen, Germany) [26].

On the 3 Tesla system DWI was performed using a single-shot spin-echo (SE) echo-planar (EPI) sequence with the following parameters: echo time (TE)/ repetition time (TR) = 86–109 ms/ 3000–8100 ms, flip angle (FA) = 90°, slice thickness (ST) = 5–6 mm, field of view (FOV) = 229 × 229 mm, echo train length (ETL) = 1, number of slices (NS) = 19–27, spacing between slices (SS) = 5–7.2 mm, acquisition matrix (matrix) = 128–130 × 128–130, number of averages (NA) = 3–4, parallel-acquisition-technique factor (PAT) = 2. Diffusion sensitizing gradients were applied sequentially in the *x*, *y*, and *z* directions with *b*-values of 0 and 1200 s/mm^2^.

On the 1.5 Tesla system DWI parameters were as followed: TE/TR = 136 ms/ 3800–4200 ms, FA = 90°, ST = 6 mm, FOV = 230 × 230 mm, ETL = 1, NS = 19–21, SS = 6.6 mm, matrix = 128 × 98, NA = 4, PAT = 2. Diffusion sensitizing gradients were applied sequentially in the *x*, *y*, and *z* directions with *b*-values of 0 and 1000 s/mm^2^.

Subsequently, post-contrast T1w magnetization-prepared rapid gradient-echo (MP-RAGE) data were acquired: Inversion time (TI) = 900–1100 ms, TE/TR = 2.66–3.57 ms/ 1740–1900 ms, FA = 9–15°, ST = 1 mm, FOV = 250–270*250–270 mm, ETL = 1, matrix = 320–384 × 201–264, NA = 1, PAT = 2 for the 3 Tesla System and TI = 1100 ms, TE/TR = 3.49 ms/ 2160 ms, FA = 15°, ST = 1.3 mm, FOV = 250*250 mm, ETL = 1, matrix = 256 × 256, NA = 1, PAT = 2 for the 1.5 Tesla system.

### Image post-processing and analysis

ADC maps were generated using in-house Siemens Syngo Software (Leonardo, Siemens Medical Systems). The purpose-built software termed Prima (DKFZ Heidelberg, Germany) based on MeVisLab (Fraunhofer MEVIS, Bremen, Germany) was used to process these maps and contrast-enhanced axial MP-RAGE data.

ADC maps and T1w images were coregistered using a linear rigid registration algorithm based on mutual information followed by visual inspection to ensure adequate alignment [27,28].

Subsequently, regions of interest (ROIs) were manually delineated on the T1w follow-up images, encompassing the enhancing lesion on the section with the largest diameter of the enhancement.

For reference, a second ROI was manually delineated within the white matter on the contralateral hemisphere. All following values were normalized to the reference ROI.

Due to co-registration both ROIs were directly transferable to the ADC maps. Thereafter, we calculated relative ADC (rADC) (baseline)/reference ROI and rADC (follow-up)/reference ROI values for each voxel within the tumor ROI.

Parametric response maps were determined as the difference between the rADC intensities between the follow-up and baseline images.

All tumor voxels were automatically segmented into three different categories and color-coded to visualize changes: red voxels for which the rADC increased significantly (ΔADC > 0.25), blue voxels for which the rADC decreased significantly (ΔADC < -0.25) and green voxels (|ΔADC| ≤ 0.25) with no significant change.

### Statistical analysis

Receiver operating characteristic curve analysis with calculation of the area under the curve (AUC) was used to determine the threshold for which a voxel’s change of the rADC values is best to differentiate between PD and PsP [29]. Four different thresholds (0.25; 0.5; 0.75 and 1) were tested to determine the most suitable one.

For each threshold the percentage of voxels within the ROI with 1) a decrease above the threshold (e.g. -0.25), 2) an increase above the threshold (e.g. 0.25), or 3) a decrease and in increase in between the thresholds (e.g. ≥ -0.25 and ≤ 0.25) was calculated. Subsequently, receiver operating characteristic curve analysis was calculated for voxels with significantly decreasing rADC values. We considered voxels with changes between the thresholds as a stable condition and combined this fraction with voxels with increasing values for further testing.

Moreover, we used a logistic regression to receive the respective *p* value for rADC performance.

The cutoff value between the two groups was considered optimal when the Youden index (sensitivity + specificity -1) reached a maximum.

We hypothesized that the amount of voxels with a rADC decrease ≥ 0.25 could differentiate between PD and PsP so we applied a Mann-Whitney U test.

For all statistical tests, the results were considered statistically significant at the two-sided significance level α < .05. All statistical computations were performed with the statistical software package SPSS 22.0, Chicago, IL.

## Results

A total of 35 patients with histopathologically proven glioblastoma were included in the analyses with 28 patients suffering from PD and seven from PsP. Regarding the patients with PD 18 had shown a new enhancement in the first, five in the second and five in the third follow-up scan. Four of the patients with PsP had shown an enhancement in the first, two in the second and one in the third follow-up scan.

Receiver operating characteristic curve analysis revealed the optimal threshold of 0.25 with an AUC of 0.844±0.065 (*p* = 0.014) to differentiate between PD and PsP. The AUC for 0.5 was 0.837±0.069 (*p* = 0.025), for 0.75 it was 0.837±0.069 (*p* = 0.054) and for 1 the AUC was 0.832±0.068 (*p* = 0.135). Hence, for further analysis we used 0.25 as a threshold.

**Fig 1:**
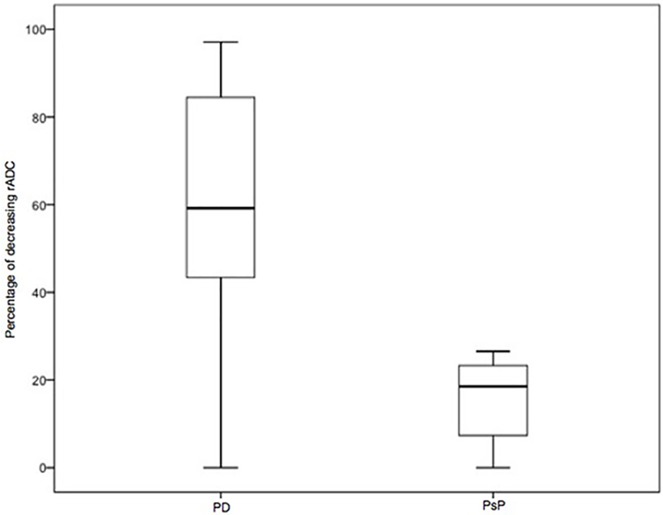
Boxplots of relative apparent diffusion coefficient (rADC) from patients with progressive disease (PD) and pseudoprogression (PsP). Boxplots showing the percentage of voxels with a decrease of rADC ≥ 0.25 between baseline and follow-up rADC values for patients with PD (n = 28) and PsP (n = 7). The percentage of rADC is higher in patients with PD (59.2%; Q1 36.7; Q3 78.6) than in those with PsP (18.6%; Q1 3.04; Q3 26.5) (*p* = 0.005).

Patients with PD showed a rADC decrease in 59.2% of all voxels (1^st^ quartile (Q1) 36.7; 3^rd^ quartile (Q3) 78.6) between the baseline and the follow-up MRI scans. Whereas in patients with PsP a decrease of only 18.6% occurred (Q1 3.04; Q3 26.5) (Fig 1).

There was a significant difference between voxels with a rADC decrease ≥ 0.25 for patients with PD and PsP (*p* = 0.005) and hence patients with PD did have a significantly higher diffusion restriction than patients with PsP.

**Fig 2:**
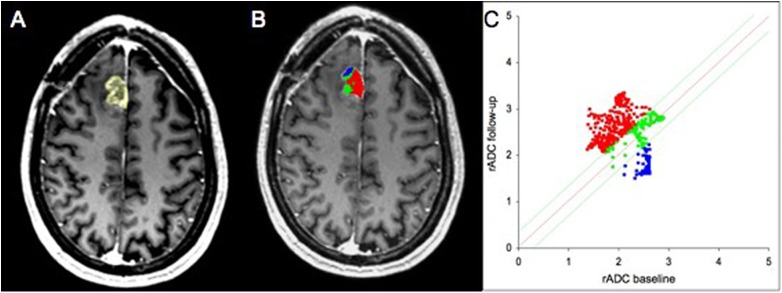
Analysis of a 49-year-old male patient with progressive disease (PD). Contrast enhanced T1w MR images (A-B). Follow-up image with delineated tumor region of interest (ROI) (A). Parametric response map of relative apparent diffusion coefficient (rADC) (B). The resulting quantitave scatter plot (C). The voxels are color-coded corresponding to their changes between baseline and follow-up examinations. Blue is designated to voxels with a decrease of rADC ≥ 0.25, red to an increase of rACD < 0.25 and green to changes in between these thresholds.

**Fig 3:**
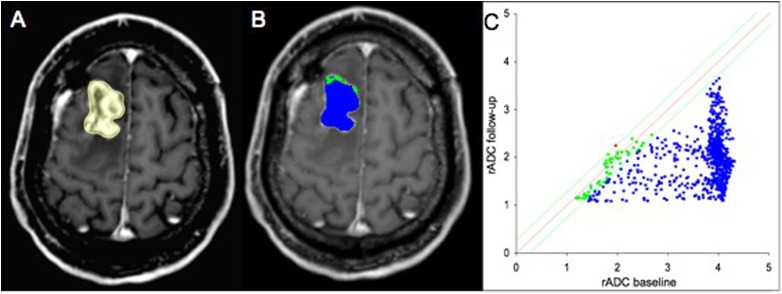
Analysis of a 38-year-old female patient with pseudoprogression (PsP). Contrast enhanced T1w MR images (A-B). Follow-up image with delineated tumor region of interest (ROI) (A). Parametric response map of relative apparent diffusion coefficient (rADC) (B). The resulting quantitave scatter plot (C). The voxels are color-coded corresponding to their changes between baseline and follow-up examinations. Blue is designated to voxels with a decrease of rADC ≥ 0.25, red to an increase of rACD < 0.25 and green to changes in between these thresholds.

The calculation of the maximum Youden index (Youden index = 0.72, sensitivity = 0.86, specificity = 0.86) revealed a percentage of voxels with a decrease of rADC of 27.05% as an optimal cutoff value between PsP and PD. Thus, if the amount of these voxels exceeds 27.05%, the patient is likely to suffer from PD. Figs 2 and 3 show the analysis of a patient with PD and a patient with PsP respectively.

## Discussion

This study has used voxel-wise changes of rADC values, calculated into parametric response maps, to distinguish between PsP and PD in glioblastoma patients following current standard treatment and follow-up imaging. We found that this method could differentiate between the two groups with a sensitivity and a specificity of 86% respectively and an AUC of 0.844±0.065 (*p* = 0.014). Notably, the diffusion restriction of patients with PD was significantly more pronounced than in patients with PsP (*p* = 0.005). Herby a potential option is given to differentiate the two phenomena. The calculated cutoff value between PsP and PD (amount of voxels with decreasing rADC of 27.05% or higher is supposed to be PD) could potentially be relevant for the determination of group affiliation and subsequently for making therapy decisions. The early diagnosis of PsP versus PD is crucial in order to tailor the best possible treatment strategies to individual patients. This pertinent question is currently being evaluated in many studies.

So far, there’s no consistency in the analysis of rADC values using parametric response maps between different research groups. Several studies investigating ADC have been suffering from limitations like heterogeneous patient groups with different tumor grades [21,30–32]. In this context it is important to note that glioblastoma with IDH 1 or IDH 2 mutation and those without are now widely regarded as different tumors [33]. Therefore the distribution of IDH mutation between patients with PD and PsP should be balanced to receive comparable results. Moreover different approaches for determining the ADC-threshold were performed among the different groups. One group used an ADC-threshold determined by empiric data of 15 patients [30–32], while others used ADC-thresholds that equaled the 95% confidence interval of a mixture of grey and white matter scans of 69 patients with different tumor grades [21].

Strengths of the current study are that all thresholds were determined after performing normalization for every single patient and examinations were done during the normal clinical routine. Therefore it is possible to transfer the method on other already existing patient groups and as a result increase the number of patients and improve statistical power. Moreover this could help to standardize the analysis process in the format of a pooled analysis or meta-analysis.

However, limitations of the current study have to be acknowledged and are mostly caused by the retrospective design of the study, the limited number of included patients and the used postprocessing algorithm.

The applied rigid co-registration is prone to inaccuracy in case of mass effects occurring between baseline and follow-up scans. Mechanisms can be significant tumor growth, high intracranial pressure due to edema, or hydrocephalus. Furthermore trepanation and opening the dura during tumor resection may cause changes in brain geometry, which is termed as brain-shift [34]. If this occurs, rigid registration does not guarantee accurate results. In our study co-registration was followed by visual inspection to ensure adequate alignment. A possible solution to this problem might be using elastic registration as proposed by Ardekani et al. [35] and evaluated by Ellingson et al. [36]. Another limitation is that defining the tumor ROI might cause problems in case of multifocal glioblastoma. A 3D-based approach may solve that problem.

An obvious limitation of the current study as well as of all studies that aim to differentiate between PD and PsP is that both entities might coexist in the same patient at the same time in different areas of the tumor. Further research is needed to determine how these cases might be best diagnosed and treated.

Another limitation of our study are varying field strengths and imaging parameters. The MR examinations of our study were acquired over a period of several years by using two scanners with 1.5 and 3 Tesla and unfortunately diffusion imaging parameters, varied in this period. Generally, ADC values are not only dependent on physiological parameters, such as temperature, restriction and perfusion but also depend on MRI sequence parameters, like b-values [20]. Future studies might use more sophisticated standardization and postprocessing-techniques to overcome this limitation [37].

It finally has to be acknowledged that our classifications based on the cutoff value of 27.05% would have led to misleading results in five patients. Four out of 28 patients with PD were mistakenly classified as PsP and one patient out of seven with PsP was classified as PD (sensitivity 0.86; specificity 0.86). No mass effects had occurred in these patients that could be a bias for the false classification.

In comparison to further studies that assessed the diagnostic potential for differentiation of PsP and PD, the results of the current study might be more promising than studies evaluating diffusion tensor imaging, dynamic susceptibility contrast and quantitative dynamic contrast-enhanced MRI [38,39]. On the other hand we received less promising results than recently published data by Galldiks et al. for PET [40]. However, it must be emphasized that the low number and vast heterogeneity of included patients in the majority of pseudoprogression-studies makes a direct comparison between the results of these studies nearly impossible.

Future studies should finally assess the introduced parametric response maps with a combined approach of a multitude of MRI and PET-techniques.

In summary, we showed that we were able to differentiate between patients with PD and PsP by using the percentage of voxels with decreasing rADC values. For widespread clinical use of this methodology, it would be necessary to standardize the process of acquiring and analyzing MRI data to receive comparable results and higher patient numbers. At present, the method studied is not robust enough to serve as an immediate exclusive tool to distinguish between PD and PsP in patients with glioblastoma receiving CRT, but may aid in the complex decision process.
